# Dual-purpose elemental sulfur for capturing and accelerating biodegradation of petroleum hydrocarbons in anaerobic environment

**DOI:** 10.1016/j.wroa.2024.100290

**Published:** 2024-12-02

**Authors:** Qian Zhao, Chengmei Liao, Enli Jiang, Xuejun Yan, Huijuan Su, Lili Tian, Nan Li, Fernanda Leite Lobo, Xin Wang

**Affiliations:** a MOE Key Laboratory of Pollution Processes and Environmental Criteria, Tianjin Key Laboratory of Environmental Remediation and Pollution Control, Nankai University, No. 38 Tongyan Road, Jinnan District, Tianjin 300350, PR China; b School of Ecology and Environment, Inner Mongolia University, Hohhot 010021, PR China; c School of Environmental and Chemical Engineering, Yanshan University, Qinhuangdao 066004, PR China; d School of Environmental Science and Engineering, Tianjin University, No. 135 Yaguan Road, Jinnan District, Tianjin 300350, PR China; e Department of Hydraulic and Environmental Engineering, Federal University of Ceará (UFC), Campus Do Pici 60.440-900, Fortaleza, CE, Brazil

**Keywords:** Petroleum hydrocarbons, Bioremediation, Elemental sulfur, Hydrophobicity, Synergistic metabolism

## Abstract

Hydrophobic organic pollutants in aqueous environments are challenging to biodegrade due to limited contact between microorganisms, the pollutants and the electron acceptor, particularly under anaerobic or anoxic conditions. Here, we propose a novel strategy that uses inexpensive, dual-function elemental sulfur (S^0^) to enhance biodegradation. Using petroleum hydrocarbons as the target pollutants, we demonstrated that hydrophobic and nonpolar S° can concentrate hydrocarbons while simultaneously serving as an electron acceptor to enrich hydrocarbon-degrading bacteria. The permeable reactive barrier filled with S^0^ effectively removed petroleum hydrocarbons. In addition to rapid adsorption, we discovered, for the first time, that petroleum hydrocarbons underwent efficient biodegradation through the reduction of S^0^. Specifically, n-alkanes were degraded by 80 % to 90 % and polycyclic aromatic hydrocarbons by 40 % to 95 %. These degradation rates were 17 % to 30 % and 26 % to 43 % higher, respectively, compared to those observed without S^0^. Consecutive subcultures combined with untargeted metabolomics analysis revealed that bacteria capable of dissimilatory sulfur reduction enhanced the fermentation process. These bacteria provided electrons to the metabolic network, which facilitated the mineralization of petroleum hydrocarbons. Our findings highlight the significant potential of S° for removing hydrophobic organic pollutants in oxygen-free environments, demonstrate the feasibility of integrating adsorption, biodegradation, and electron supply to enhance pollutant removal.

## Highlights

• Hydrophobic and nonpolar elemental sulfur (S^0^) can capture petroleum hydrocarbons.
• S^0^ acted as an electron acceptor, enriching hydrocarbon-degrading bacteria.
• S^0^ enhanced petroleum hydrocarbon biodegradation more effectively than without S^0^.
• Incorporating dissimilatory sulfur reduction improved the fermentation efficiency.

## Graphical abstract

**Figure:**
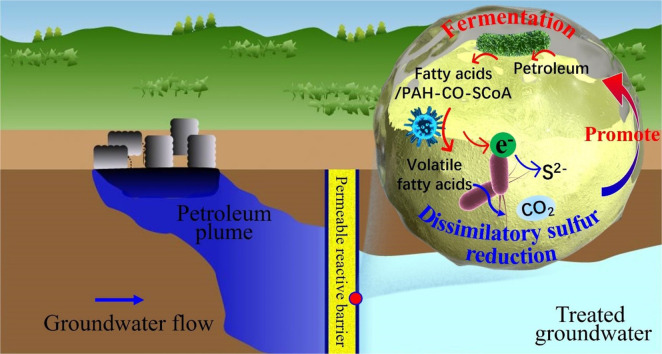

## Introduction

Hydrophobic organic pollutants are often difficult to biodegrade in aquatic environments, primarily due to the poor interaction between the electron donors (pollutants), the catalysts (microbes) and the electron acceptors. Petroleum hydrocarbons are representative hydrophobic pollutants. Due to their high C/N ratio, long carbon chains, water insolubility, low polarity, potential for bioaccumulation, harmful effects, persistence and complex nature (Wang et al., 2020), petroleum hydrocarbons pose a serious threat to human health and ecosystems when leaked into the environment (Varjani and Upasani, 2017). Biological degradation is a green and crucial process for the remediation of these pollutants (Varjani, 2017); however, its efficiency largely depends on the availability of both the pollutants and the electron acceptors for the degrading microbes, especially in anaerobic conditions such as groundwater or anoxic zone in water bodies.

In anaerobic environments, microorganisms can use nitrate, sulfate and metal oxides as alternative electron acceptors to facilitate the degradation of petroleum hydrocarbons (Sajid et al., 2023; Tang et al., 2021; Zhang et al., 2015). Concentrating hydrophobic pollutants and alternative electron acceptors near microorganisms is believed to enhance biodegradation in these environments. Given this, can the strategic selection of electron acceptors achieve both goals simultaneously? We propose that elemental sulfur (S^0^) is an ideal choice, because it is a nonpolar solid that can accumulate hydrocarbons in water and is biologically reducible.

S^0^ is a major form of sulfur element in the Earth's crust, which exists in a solid state at room temperature. It is nonpolar and hydrophobic, and can be reduced to sulfide by microorganisms, which then precipitates with heavy metals during wastewater treatment (Florentino et al., 2016; Zhang et al., 2018a). It has been reported that various microorganisms, including *Wolinella, Pseudomonas, Geobacter, Desulfuromonas, Desulfuromusa, Desulfovibrio* and *Desulfoicroum* (Hedderich et al., 1998; Rabus et al., 2006), can oxidize organics such as acetate, sugar, lactic acid, ethanol and peptone, coupled with dissimilatory sulfur reduction to sulfide. For example, the reaction between acetate and S^0^, shown in eq. (1), releases 39 kJ/mol of energy, showing the possibility of bioenergy gain through the degradation. This reduction can be further accelerated by self-produced polysulfides (Zhang et al., 2018b). Currently, it is unclear whether the biological reduction of S° can be effectively coupled with the degradation of petroleum hydrocarbons.

(1) $$CH_{3}COO^{-}+4S^{0}+H^{+}+2H_{2}O\to 2CO_{2}+4H_{2}S\Delta G^{0}=-39\;kJ\cdot mol^{-1}$$

Here the nonpolar and hydrophobic S^0^ was designed to capture petroleum hydrocarbons from water, brought the distance between the electron donor and electron acceptor closer together spatially, and then the microbial community was acclimated at the interface of oil-sulfur. This was demonstrated by using a permeable reactive barrier (PRB) where S^0^ particles served as a bio-reactive medium to remove petroleum hydrocarbons. Batch experiments were carried out to further investigate the involvement of S^0^ in the biodegradation process, and to explore the underlying mechanisms of microbial metabolism.

## Results and discussion

### S° captured petroleum hydrocarbons in PRB

**Fig. 1:**
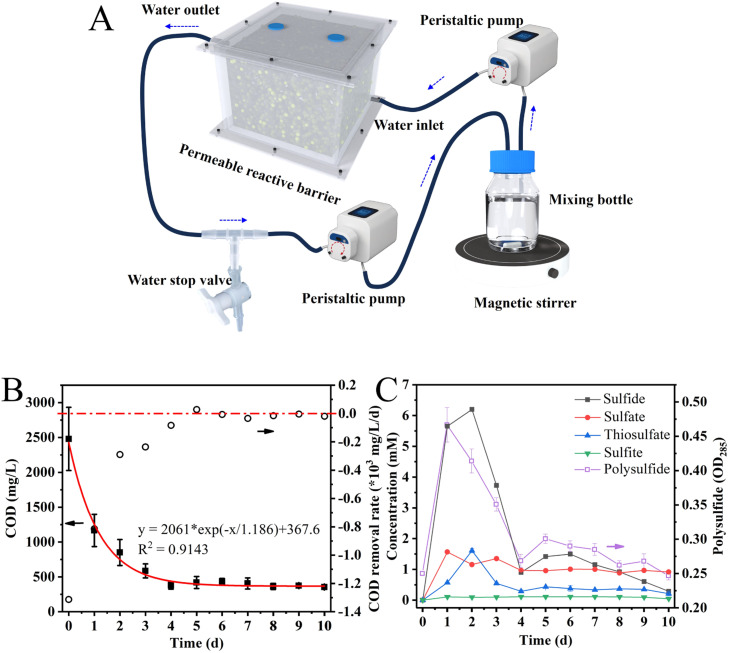
Schematic representation of the PRB reactor (A). The arrow indicates the direction of fluid flow. COD concentration and COD removal rate (B), sulfide and sulfite concentration, polysulfide, sulfate, and thiosulfate concentration varied with time in the PRB reactor (C).

PRB reactors filled with S^0^ particles were constructed to demonstrate the effectiveness of S^0^ as a reactive medium for the removal of petroleum hydrocarbons. (Fig. 1**A**) After three cycles of acclimation, a significant reduction of COD was observed, with a 53 % decrease on the first day, from 2480 ± 452 mg/L to 1166 ± 232 mg/L, and an 85 % reduction to 378 ± 53 mg/L by the fourth day (Fig. 1**B**). Following this rapid removal phase, the COD maintained a low average removal rate of 16 mg/L/d (Fig. 1**B**). The rapid removal in COD during the initial phase can be preliminarily attributed to the adsorption of petroleum hydrocarbon onto S^0^ particles. The kinetic modeling indicated that the variation in COD concentration over time followed a pseudo-first-order rate equation, consistent with the adsorption kinetics (Qiu et al., 2009). The sulfate, thiosulfate and sulfite concentrations remained low, below 1 mM throughout the process (Fig. 1**C**). Interestingly, the cumulative concentrations of sulfide and polysulfide sharply increased in the initial 4 days, suggesting that S^0^ was continuously reduced in the rapid removal period. Therefore, we deduced that the oxidation of petroleum hydrocarbons may occur simultaneously, probably powered by microorganisms. To clarify the reactions occurring during this period, batch investigations were conducted in serum bottles.

### S^0^ is a bioavailable electron acceptor for petroleum hydrocarbon degradation

**Fig. 2:**
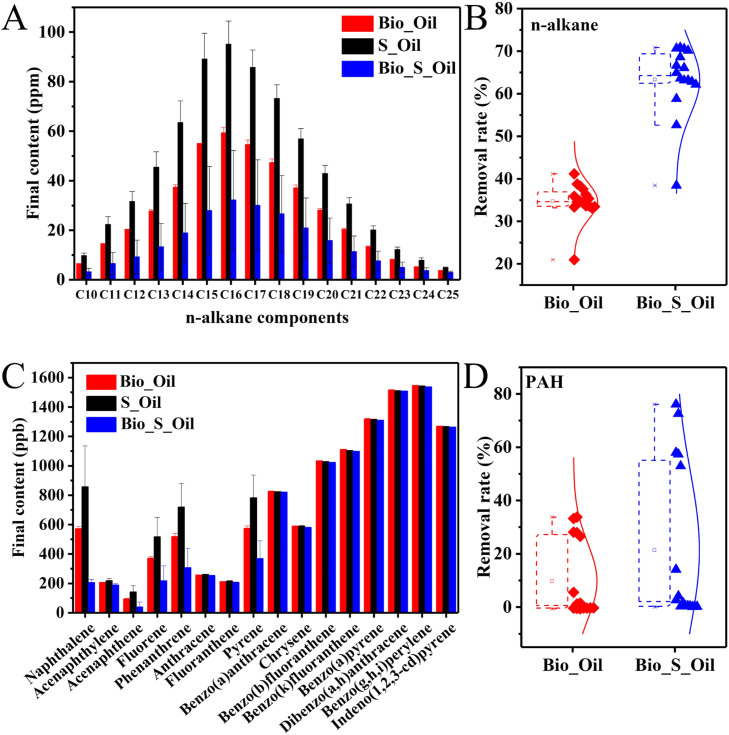
The components of alkanes (A) and PAHs (C) in Bio_Oil, S_Oil and Bio_S_Oil after 90 days. The overall degradation efficiency of alkanes (B) and PAHs (D) in Bio_Oil and Bio_S_Oil.

Petroleum hydrocarbons are hydrophobic and typically nonpolar or weakly polar, similar to S^0^. These shared characteristics facilitate their close adsorption to each other in water. To assess the impact of S^0^ on the biodegradation of petroleum hydrocarbons, three groups were compared: the biological group without S^0^ (Bio_Oil), the sterile S^0^ group (S_Oil) and the biological S^0^ group (Bio_S_Oil) (Fig. 2). The degradation efficiency of n-alkanes in the biological S^0^ group ranged from 38 % to 71 %, with a value of 17 % to 30 % higher than that observed in the absence of S^0^, demonstrating that S^0^ was an available electron acceptor accelerating biological hydrocarbon oxidation (Figs. 2**A and** 2**B**). The presence of S^0^ enhanced the biological mineralization of alkanes or their fermentation products. However, the degradation of long carbon chain alkanes, such as C25, was relatively low with 21 % in the Bio_Oil, and the addition of S^0^ resulted in the lowest improvement of 17 % in degradation efficiency.

PAHs including naphthalene, acenaphthene, pyrene, fluorene, and phenanthrene were degraded in both biological groups (Bio_Oil and Bio_S_Oil) than the sterile S^0^ group (Figs. 2**C and** 2**D**), achieving degradation efficiencies of 26 %–76 %, demonstrating that the biodegradation also contributed to the removal of toxic PAHs. In the presence of S^0^ (Bio_S_Oil), the biodegradation efficiencies of naphthalene, acenaphthene, pyrene, fluorene and phenanthrene increased by 26 %–43 % compared to those in the Bio_Oil group, with an overall improvement of 15 %. This suggested that the additional electron acceptor, S^0^, accelerated the degradation of certain low molecular weight PAHs, similar to the effect observed with alkanes. Likewise, high molecular weight PAHs, such as anthracene, fluoranthene, benzo[a]anthracene, chrysene, benzo[b]fluoranthene, benzo[k]fluoranthene, benzo[a]pyrene, dibenzo[a, h]anthracene, benzo[g, h, i]perylene and indeno[1, 2, 3-cd]pyrene were not able to be biodegraded in the tested period, regardless of whether S^0^ was added or not. This may be attributed to their relatively complex structure and biotoxicity (Ghosal et al., 2016). According to literatures, a longer enrichment period or the addition of functional bacteria could improve their degradation (Boonchan et al., 2000; Juhasz and Naidu, 2000).

**Fig. 3:**
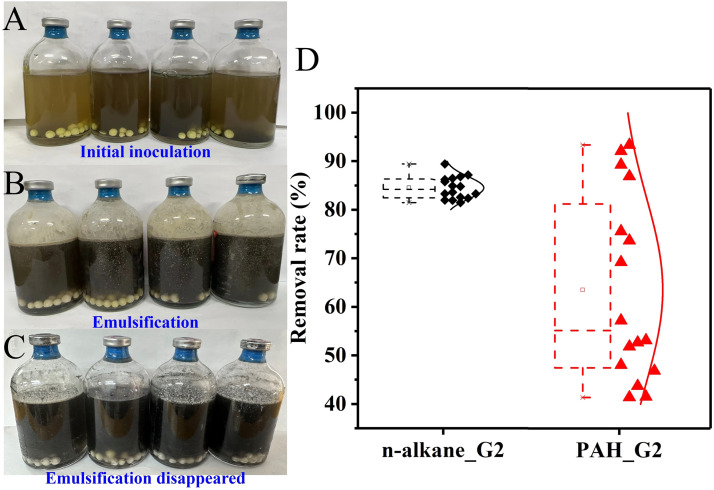
The images show the initial inoculation (A), emulsification on day 3 (B) and de-emulsification on day 12 (C) of the second generation in batch culture of serum bottles. Alkanes and PAHs degradation efficiencies (D) after 108 days of cultivation.

Subcultivation of the Bio_S_Oil demonstrated that petroleum hydrocarbon biodegradation using S^0^ was repeatable, with significantly improved degradation performance observed in the second generation. Initially, the diesel injected formed an oil layer on the surface of the culture medium (Fig. 3**A**). Emulsification occurred on the third day after inoculation, appearing as a layer of white, milky turbidity on the bottle wall (Fig. 3**B**). The emulsified oil-sulfur mixture was suspended and uniformly dispersed throughout the medium until the 12th day (Figs. 3**C and S1**), indicating that some metabolites produced by microorganisms had surfactant properties, which increased the availability of petroleum hydrocarbons to microorganisms (Eftaxias et al., 2021). The entire suspended oil-sulfur mixture had disappeared on day 12, indicating that these hydrophobic pollutants were converted into soluble substances, thereby facilitating subsequent biodegradation. The third generation exhibited a similar phenomenon to the second. After 108 days of cultivation, the degradation rates of alkanes (C10–C25) and PAHs reached 80 %–90 % and 40 %–95 % (Fig. 3**D**). Among the 16 PAHs, fluoranthene and naphthalene exhibited the highest degradation efficiencies at 93 % and 92 %, respectively. Acenaphthylene and acenaphthene followed with 89 % and 87 %, while phenanthrene, anthracene, and fluorene demonstrated moderate efficiencies of 76 %, 74 % and 69 %. The remaining 9 PAHs showed the lowest degradation efficiencies, ranging from 40 % to 50 %. Nevertheless, the overall biodegradation efficiency of PAHs with S^0^ substantially improved from 15 % (first generation) to 71 % (second generation) after subcultivation, showing the evolution of the microbial community. Sulfide accumulated over the 108 days of cultivation, with its concentration increasing 25-fold from 0.26 ± 0.03 mM to 6.5 ± 0.9 mM. Meanwhile, the concentrations of sulfate and thiosulfate increased by 2-fold and 31-fold, indicating simultaneous S^0^ reduction and disproportionation reactions.

### The microbial community degrading petroleum hydrocarbon using S^0^

To understand the role of S^0^, the microbial communities were compared in its presence (Bio_S_Oil) and absence (Bio_Oil) (**Fig. S2**). Our findings revealed that S^0^ induced the growth of certain bacteria associated with S^0^ reduction, such as *Desulfobulbus* and *Desulfurmonas*, whose abundance was 22 % higher than that in the Bio_Oil group (**Figs. S2Aand S2B**). *Desulfobulbus* has been reported to break down complex organic compounds, while the genome of *Desulfuromonas* sp. exhibits metabolic versatility in degrading aromatic hydrocarbons and utilizing various electron acceptors (Ambaye et al., 2023; Castro et al., 2022; Zhang et al., 2021). Furthermore, S^0^ significantly enhanced the microbial function of hydrocarbon degradation, iron respiration and processes related to sulfur respiration, including sulfur respiration, sulfite respiration, sulfate respiration, thiosulfate respiration and the respiration of sulfur compounds (*P* < 0.05) (**Fig. S2C**). These observations provide solid evidence that S^0^ is involved in biological petroleum hydrocarbon degradation, aligning with the efficient biodegradation performance results.

**Fig. 4:**
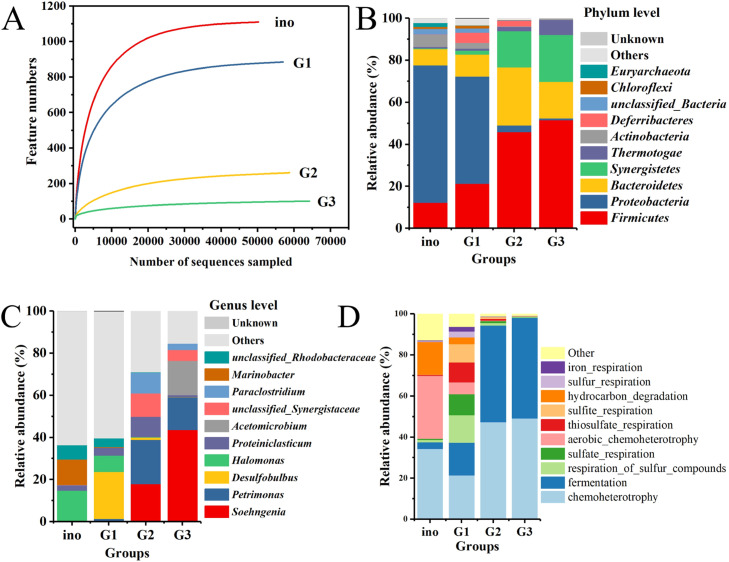
Rarefaction curve for microbial population under the serial subcultivation of elemental sulfur (A). Microbial communities in samples from the inoculation source, as well as from the first, second and third generations during serial subcultivation with elemental sulfur, analyzed at the phylum level (B) and genus level (C), and microbial functional prediction analysis (D). The labels of “ino”, “G1”, “G2” and “G3” represent the microbial samples from inoculation source, the first, second and third generation, respectively.

The composition of microbial community during the two successive subcultivations showed a clear evolution (Fig. 4). The rarefaction curve for each sample gradually leveled off with sequencing depth, indicating that the sequencing depth used in this experiment was appropriate and sufficient to capture information on most microbial species in the samples. Compared to the initial inoculum (ino), the number of features steadily decreased with subcultivation, suggesting that the microbiome continually adapted to the acclimation conditions of S^0^ (Fig. 4**A**). The composition of microbial community varied after serial subcultivations in the presence of S^0^, with the abundance of Firmicutes, Bacteroidetes, Synergistetes and Thermotogae phyla increasing after each subcultivation, reaching 52 %, 17 %, 22 % and 7 % by the end of the third generation (Fig. 4**B**). These phyla are closely associated with the fermentation and degradation of petroleum hydrocarbons (Koshlaf et al., 2019; Liu et al., 2021; Rajbongshi and Gogoi, 2021; Zhao et al., 2022). At the genus level, there was a significant enrichment of the hydrolytic bacterium *Soehngenia*, which can reduce inorganic sulfur compounds such as thiosulfate and S^0^ (Alauzet et al., 2014; Xu et al., 2023). Its relative abundance increased from 20 % to 44 % between the second and third generations (Fig. 4**C**). Additionally, the anaerobic S^0^ reducer *Petrimonas* accounted for a substantial proportion, ranging from 15 % to 21 % (Fig. 4**C**). It is able to ferment organic acids and polysaccharides to produce CO_2_, H_2_ and acetate (Chen et al., 2023; Huang et al., 2020). *Petrimonas* has been reported to possess a large number of genes encoding enzymes that catalyze the degradation of PAH intermediate metabolites (Qian et al., 2021). Furthermore, *Acetomicrobium, Paraclostridium* and *Proteiniclasticum* also experienced enrichment and played important roles in hydrolysis and short-chain fatty acid production (Fig. 4**C**) (Wang et al., 2023). Consequently, in addition to sulfur compound-related respiration processes, both chemoheterotrophy and fermentation showed a notable increase of 1.4-fold and 15-fold respectively after three generations compared to the initial inoculum, accounting for over 98 % of the total abundance (Fig. 4**D**). These results indicated the microbial community changed directionally due to the influence of S^0^.

### Degradation strategy of petroleum hydrocarbons with S^0^

**Fig. 5:**
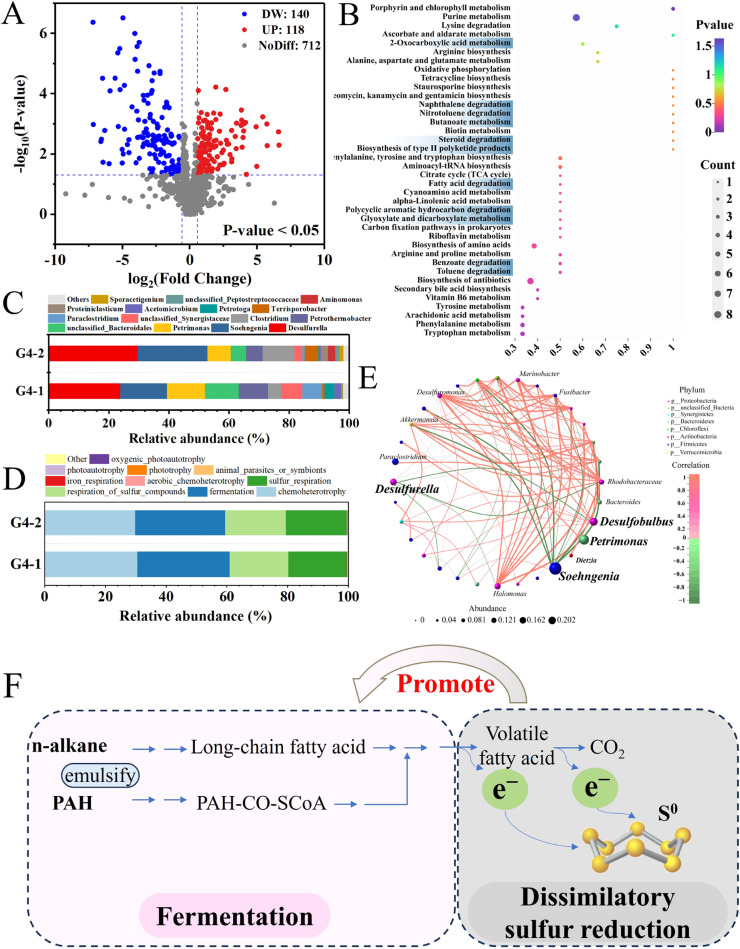
Volcanic map of differential metabolites in the fourth-generation culture (A) comparing culture for 17 days to initial inoculation (PS17 vs. PS0). KEGG pathway enrichment analysis (B). The blue highlights metabolic pathways associated with the degradation of petroleum hydrocarbons and their secondary metabolites. Microbial community composition (top 10 genera) (C) and microbial functional prediction analysis (D) for the fourth generation. Labels “G4–1″ and “G4–2″ denote duplicates of the fourth generation. Microbial correlation network diagram (E). Mechanism diagram of S^0^ reduction achieving efficient biodegradation of petroleum hydrocarbons (F).

Non-targeted metabolomics analysis was further employed to explore the metabolic strategy for petroleum hydrocarbons in the presence of S^0^ in the fourth generation. Among 970 metabolites annotated after 17 days, 140 were down-regulated, 118 were up-regulated, and 712 showed no significant change compared to the initial levels (Fig. 5**A**). The KEGG pathway analysis showed that metabolic pathways associated with the degradation of petroleum hydrocarbons (including PAH degradation, toluene degradation and naphthalene degradation) and secondary metabolites of petroleum hydrocarbons (including fatty acid degradation, 2-oxocarboxylic acid metabolism, nitrotoluene degradation, butanoate degradation, steroid degradation and benzoate degradation) were significantly enriched (Fig. 5**B**), consistent with the observed degradation performance in the presence of S^0^. Furthermore, numerous amino acid metabolism pathways (e.g., lysine degradation, arginine biosynthesis, phenylalanine metabolism, tryptophan metabolism) were also enriched, suggesting the stable growth and robust metabolic activity of the microorganisms. Additionally, the TCA cycle, which is involved in energy production, was also showed enrichment, indicating the potential for complete mineralization of petroleum hydrocarbons into CO₂.

*Soehngenia* from hydrolytic bacteria and *Desulfurella* from sulfur-reducing bacteria (S^0^RB) predominated in the fourth generation, constituting 40 % to 50 % of the total abundance, as shown in Fig. 5**C**. *Soehngenia* was selectively enriched in the previous subcultures (second and third generations), reaching the maximum abundance of ∼40 % at the end of the third generation. Its abundance remained high at 19 % in the next generation, highlighting the significance of bacteria from this genus. *Desulfurella* can utilize organic acids (Guo et al., 2022) and short-chain fatty acids (Florentino et al., 2017) and is known for its efficient dissimilatory sulfur reduction. It increased to 27 % in the fourth generation alongside *Soehngenia*, indicating a potential cooperation between hydrolytic bacteria and S^0^RB.

Although many microorganisms were not abundant, they established a range of symbiotic and competitive relationships with the dominant species (Fig. 5**E**). Fermentative species (e.g., *Soehngenia, Petrimonas, Acetomicrobium*) generated low-molecular-weight organic compounds (e.g., volatile fatty acids produced from long-chain fatty acid) through metabolic pathways associated with the degradation of petroleum hydrocarbons and secondary metabolites of petroleum hydrocarbons (Fig. 5**B**), which were used by the S^0^RB (e.g., *Desulfurella*) as the major electron donors. The electrons generated through chemoheterotrophy and fermentation were utilized in sulfur compound respiration and sulfur respiration (Fig. 5**D**), process known as dissimilatory sulfur reduction. These activities further facilitated the fermentation process and supported the emulsification and complete mineralization of secondary metabolites of petroleum hydrocarbons (Fig. 5**F**), as demonstrated by the degradation performance (Figs. 2 **and** 3).

## Conclusions

The degradation of hydrophobic organic pollutants is difficult in anaerobic or anoxic environment. Here, we demonstrated that S^0^ served dual roles as both an adsorbent and an electron acceptor, effectively reducing the distance between pollutants, microorganisms and the electron acceptor. Rapid adsorption occurred based on the principle of "like dissolves like," with petroleum hydrocarbons undergoing efficient biodegradation (80 %∼90 % for n-alkanes and 40 %∼95 % for PAHs) when using hydrophobic S^0^ as the sole electron acceptor. Dissimilatory sulfur reduction was found to enhance the fermentation process by supplying electrons, which, in turn, facilitated the further mineralization of petroleum hydrocarbons.

It is both simple and cost-effective to scale up in reactors such as PRBs, as preliminarily tested in our study, for removing petroleum hydrocarbons from groundwater contaminated by gas tank leaks. Although the use of elemental sulfur for subsurface remediation may result in malodorous sulfide-containing water, the resulting sulfides can be removed by adding metal oxides to the packing materials, or by reacting with metal ions in the groundwater, which generate metal sulfide precipitates (Liu et al., 2023). Alternatively, sulfides can be removed by anodic sulfide oxidation in electrochemical/bio-electrochemical systems with a recovery production of elemental sulfur (Zhao et al., 2023). This approach is also effective for bioremediating marine areas affected by oil spills. Our findings demonstrated the potential of using S^0^ as a dual-purpose material in the bioremediation of hydrophobic pollutants, particularly in situations where electron acceptors are limited, offering a novel perspective on the selection of bioreactor packing materials. Furthermore, the S^0^-technology can also be applied to petroleum-contaminated soil remediation in the future.

## Materials and methods

### The operation of permeable reactive barrier and the inoculum

A series of PRB reactors were developed to test the removal of petroleum hydrocarbons by S^0^ (Fig. 1**A**). The main body consisted of a cubic acrylic reactor (15 cm × 10 cm × 10 cm), sealed with rubber gaskets and screws, and filled with S^0^ particles (hemisphere, 2 mm in diameter). The initial inoculum was derived from petroleum-contaminated soil collected from the Shengli oil field (118.75°E, 37.85°N, Shandong province, China). Detailed information is in **Section S1 and S2** of the Supporting Information. The medium was 50 mM phosphate buffer solution (PBS, **Table S1**) containing 12.5 mL/L trace element solution (**Table S2**), 5 mL/L vitamin solution (**Table S3**), 0.05 % yeast extract and 0.05 % tryptone, which were necessary nutrients for microbial growth (Bonch-Osmolovskaya et al., 1990; Pfennig and Biebl, 1976). It was deoxygenated with N_2_/CO_2_ (80 %/20 %, V/V) before being transferred to the reactors, and 2 mL of diesel oil (0#) was added to the mixing bottle (250 mL) as the contaminated water sample. It was pumped through the reactor at a flow rate of 50 mL/min, recirculated at 37 ± 0.5 °C, and the contaminated water sample was refreshed every 30 days, marked as a cycle. When the sulfide concentration reached 3 mM, it was removed from the system by blowing N_2_/CO_2_ (80 %/20 %, V/V) for 30 min to reduce the concentration below 0.6 mM. After two complete cycles, the diesel oil was reduced to 1 mL per cycle to test the removal efficiency. Samples were periodically collected to measure chemical oxygen demand (COD), sulfide, polysulfide, sulfate, thiosulfate and sulfite.

The microorganisms in these reactors were synchronously enriched in 100 mL anaerobic serum bottles containing the same medium. To each bottle, 3 g of S^0^ particles and 1 mL of diesel oil per 70 mL of medium were added. The microorganisms were transferred to fresh medium every 30 days to complete subculturing. Meanwhile, the medium with autoclaved soil extract (killed) and in the absence of S^0^ were simultaneously incubated as controls. All groups were prepared in triplicate and cultured in a constant temperature shaker (180 rpm, 37 ± 0.5 °C). The suspensions were collected regularly for subsequent analyses.

### Chemical analysis

Petroleum hydrocarbons were extracted from the entire enrichment medium using isopyknic dichloromethane. The detailed extraction procedures are documented in **Section S3** of the Supporting Information. The concentration of n-alkanes (C10-C25) and 16 priority control PAHs were determined by gas chromatography-mass spectrometry (GC–MS) (Zhang et al., 2022). The concentrations of sulfide, sulfate, thiosulfate, sulfite, polysulfide and COD were analyzed following filtration through a 0.22 μm pore size filter. COD was quantified by the potassium dichromate method using a COD detector (5B-1F (V8), Lianhua Technology, China). The total dissolved sulfide (H_2_S, HS^−^ and S^2−^) of the solution was analyzed at 665 nm with Multiscan Spectrum (Spark 10 M, TECAN, Switzerland) using the methylene blue spectrophotometric method with N, N-dimethylphenylenediamine as the colorimetric agent (Li et al., 2021). Sulfate was determined by the absorbance of the resulting suspension at 420 nm with barium chloride using the turbidimetric method (Kolmert et al., 2000). Thiosulfate was examined by its oxidation with iodate, the produced iodine, equivalent to the excess iodate, was measured spectrophotometrically as triiodide at 350 nm (Koh et al., 1991). Sulfite (SO_3_^2−^) was determined by its reaction with o-phthaldialdehyde in the presence of ammonia with an excitation/emission wavelength of 320/390 nm (Mana and Spohn, 2001). The concentration of polysulfide was measured at a wavelength of 285 nm (Sun et al., 2018). Each sample was tested in triplicate, and the mean value was used in calculation.

### Biological analysis

The biomass was harvested by centrifuging the samples collected from the suspension for microbial community analysis. Genomic DNA was extracted using Soil Genomic DNA Kit (CW2091S, ComWin Biotech Co. Ltd., Beijing, China). The microbial consortia analysis was performed using the Illumina MiSeq sequencing platform through BMKCloud (www.biocloud.net). The hypervariable V4 region of the 16S rRNA gene was amplified with the forward primer 515F (5′-GTGYCAGCMGCCGCGGTAA-3′) and the reverse primer 806R2 (5′-GGACTACNVGGGTWTCTAAT-3′) as universal primers. Amplicon sequence variants (ASVs) were acquired by denoising sequences using the DADA2 method in QIIME2 2020.6 software with a conservative filtration threshold of 0.005 %. The feature sequences were annotated by the Naive Bayes classifier against the NCBI reference database at various taxonomic levels (phylum, class, order, family, genus, species). Community structure maps at different taxonomic levels were generated using R language tools.

Untargeted metabolomics analysis was conducted to identify the microbial metabolites of petroleum hydrocarbons using liquid chromatography-mass spectrometry (LC-MS) by Novogene (Beijing, China). Two milliliters of suspension sample were centrifuged at 3000 rpm for 10 min at 4 °C. The supernatant liquid was quickly frozen in liquid nitrogen for 15 min and stored at −80 °C for subsequent analysis. The raw mass spectrometry data was imported into Compound Discoverer 3.3 (CD3.3) software for spectrum processing and database search (https://www.mzcloud.org/) to obtain the qualitative and quantitative results of metabolites. The KEGG database (https://www.genome.jp/kegg/pathway.html), HMDB database (https://hmdb.ca/metabolites) and LIPIDMaps database (http://www.lipidmaps.org/) were used to identify the metabolites. Differential metabolites were filtered based on Variable Importance in the Projection (VIP) > 1.0, Fold Change (FC) > 1.2 or FC < 0.833 and P-value< 0.05. Significantly enriched Kyoto Encyclopedia of Genes and Genomes (KEGG) pathways (padj < 0.05) and Receiver Operating Characteristics (ROC) were selected for further analyses using the Novamagic platform.

## CRediT authorship contribution statement

**Qian Zhao:** Writing – original draft, Visualization, Validation, Software, Methodology, Investigation, Formal analysis, Data curation. **Chengmei Liao:** Validation, Methodology, Investigation, Formal analysis, Data curation. **Enli Jiang:** Methodology, Data curation. **Xuejun Yan:** Methodology, Formal analysis. **Huijuan Su:** Methodology, Formal analysis. **Lili Tian:** Methodology, Formal analysis. **Nan Li:** Writing – review & editing. **Fernanda Leite Lobo:** Methodology. **Xin Wang:** Writing – review & editing, Validation, Supervision, Project administration, Methodology, Investigation, Funding acquisition, Conceptualization.

## Declaration of competing interest

The authors declare that they have no known competing financial interests or personal relationships that could have appeared to influence the work reported in this paper.

## Data Availability

The data that support the findings are available from the authors upon reasonable request.

## Appendix Supplementary materials

Image, application 1

## References

[bib0001] Alauzet C., Marchandin H., Courtin P., Mory F., Lemée L., Pons J.L., Chapot-Chartier M.P., Lozniewski A., Jumas-Bilak E. (2014). Multilocus analysis reveals diversity in the genus Tissierella: description of *Tissierella carlieri sp. nov.* in the new class *Tissierellia classis nov*. Syst. Appl. Microbiol..

[bib0002] Ambaye T.G., Vaccari M., Franzetti A., Prasad S., Formicola F., Rosatelli A., Hassani A., Aminabhavi T.M., Rtimi S. (2023). Microbial electrochemical bioremediation of petroleum hydrocarbons (PHCs) pollution: recent advances and outlook. Chem. Eng. J..

[bib0003] Bonch-Osmolovskaya E., Sokolova T., Kostrikina N., Zavarzin G. (1990). *Desulfurella acetivorans* gen. nov. and sp. nov.—A new thermophilic sulfur-reducing eubacterium. Arch. Microbiol..

[bib0004] Boonchan S., Britz M.L., Stanley G.A. (2000). Degradation and mineralization of high-molecular-weight polycyclic aromatic hydrocarbons by defined fungal-bacterial cocultures. Appl. Environ. Microbiol..

[bib0005] Castro A.R., Martins G., Salvador A.F., Cavaleiro A.J. (2022). Iron compounds in anaerobic degradation of petroleum hydrocarbons: a review. Microorganisms.

[bib0006] Chen R., Bao Y.X., Zhang Y.J. (2023). A review of biogenic coalbed methane experimental studies in China. Microorganisms.

[bib0007] Eftaxias A., Diamantis V., Michailidis C., Stamatelatou K., Aivasidis A. (2021). The role of emulsification as pre-treatment on the anaerobic digestion of oleic acid: process performance, modeling, and sludge metabolic properties. Biomass Convers. Biorefin..

[bib0008] Florentino A.P., Stams A.J.M., Sánchez-Andrea I. (2017). Genome sequence of *Desulfurella amilsii* strain TR1 and comparative genomics of *Desulfurellaceae* Family. Front. Microbiol..

[bib0009] Florentino, A.P., Weijma, J., Stams, A.J.M., Sanchez-Andrea, I., (2016) Biotechnology of extremophiles: advances and challenges. Rampelotto, P.H. (ed), pp. 141–175.

[bib0010] Ghosal D., Ghosh S., Dutta T.K., Ahn Y. (2016). Current state of knowledge in microbial degradation of polycyclic aromatic hydrocarbons (PAHs): a review. Front. Microbiol..

[bib0011] Guo G., Li Z.L., Chen L., Ling Q.S., Zan F.X., Isawi H., Hao T.W., Ma J., Wang Z.P., Chen G.H., Lu H. (2022). Advances in elemental sulfur-driven bioprocesses for wastewater treatment: from metabolic study to application. Water Res.

[bib0012] Hedderich R., Klimmek O., Kröger A., Dirmeier R., Keller M., Stetter K.O. (1998). Anaerobic respiration with elemental sulfur and with disulfides. FEMS Microbiol. Rev..

[bib0013] Huang X.D., Liu X.R., Chen F., Wang Y.L., Li X.M., Wang D.B., Tao Z.L.T., Xu D., Xue W.J., Geng M.Y., Yang Q. (2020). Clarithromycin affect methane production from anaerobic digestion of waste activated sludge. J. Clean. Prod..

[bib0014] Juhasz A.L., Naidu R. (2000). Bioremediation of high molecular weight polycyclic aromatic hydrocarbons: a review of the microbial degradation of benzo[a]pyrene. Int. Biodeter. Biodegr..

[bib0015] Koh T., Kitami K., Yonemura Y. (1991). Spectrophotometric determination of thiosulfate by its oxidation with iodate. Anal. Sci..

[bib0016] Kolmert Å., Wikström P., Hallberg K.B. (2000). A fast and simple turbidimetric method for the determination of sulfate in sulfate-reducing bacterial cultures. J. Microbiol. Methods..

[bib0017] Koshlaf E., Shahsavari E., Haleyur N., Osborn A.M., Ball A.S. (2019). Effect of biostimulation on the distribution and composition of the microbial community of a polycyclic aromatic hydrocarbon-contaminated landfill soil during bioremediation. Geoderma.

[bib0018] Li G.B., Liang Z.S., Sun J.L., Qiu Y.Y., Qiu C.Y., Liang X.M., Zhu Y.H., Wang P., Li Y., Jiang F. (2021). A pilot-scale sulfur-based sulfidogenic system for the treatment of Cu-laden electroplating wastewater using real domestic sewage as electron donor. Water Res.

[bib0019] Liu Y., Zhao Q., Liao C.M., Tian L.L., Yan X.J., Li N., Wang X. (2023). Anaerobic bioreduction of elemental sulfur improves bioavailability of Fe(III) oxides for bioremediation. Sci. Total. Environ..

[bib0020] Liu Y.F., Liu Z.L., Ye Y.L., Zhou L., Liu J.F., Yang S.Z., Gu J.D., Mu B.Z. (2021). *Aminirod propionatiphilus gen. nov., sp. nov.*, an isolated secondary fermenter in methanogenic hydrocarbon-degrading communities. Int. Biodeter. Biodegr..

[bib0021] Mana H., Spohn U. (2001). Sensitive and selective flow injection analysis of hydrogen sulfite/sulfur dioxide by fluorescence detection with and without membrane separation by gas diffusion. Anal. Chem..

[bib0022] Pfennig N., Biebl H. (1976). *Desulfuromonas acetoxidans* gen. nov. and sp. nov., a new anaerobic, sulfur-reducing, acetate-oxidizing bacterium. Arch. Microbiol..

[bib0023] Qian Y.F., Xu M.Y., Deng T.C., Hu W.Z., He Z.L., Yang X.A., Wang B., Song D., Chen L.T., Huang Y.D., Sun G.P. (2021). Synergistic interactions of *Desulfovibrio* and *Petrimonas* for sulfate-reduction coupling polycyclic aromatic hydrocarbon degradation. J. Hazard. Mater..

[bib0024] Qiu H., Lv L., Pan B.C., Zhang Q.J., Zhang W.M., Zhang Q.X. (2009). Critical review in adsorption kinetic models. J. Zhejiang Univ.*Sc*. A..

[bib0025] Rabus, R., Hansen, T.A., Widdel, F., (2006) Prokaryotes: a handbook on the biology of bacteria, Vol 2, 3rd Edition: Ecophysiology and biochemistry. Dworkin, M., Falkow, S., Rosenberg, E., Schleifer, K.H. and Stackebrandt, E. (eds), pp. 659–768.

[bib0026] Rajbongshi A., Gogoi S.B. (2021). A review on anaerobic microorganisms isolated from oil reservoirs. World J. Microb. Biot..

[bib0027] Sajid S., de Dios V.R., Zveushe O.K., Nabi F., Shen S., Kang Q., Zhou L., Ma L., Zhang W., Zhao Y., Han Y., Dong F. (2023). Newly isolated halotolerant Aspergillus sp. showed high diesel degradation efficiency under high salinity environment aided with hematite. J. Hazard. Mater..

[bib0028] Sun R.R., Zhang L., Zhang Z.F., Chen G.H., Jiang F. (2018). Realizing high-rate sulfur reduction under sulfate-rich conditions in a biological sulfide production system to treat metal-laden wastewater deficient in organic matter. Water Res.

[bib0029] Tang Y., Li Y., Zhang M., Xiong P., Liu L., Bao Y., Zhao Z. (2021). Link between characteristics of Fe(III) oxides and critical role in enhancing anaerobic methanogenic degradation of complex organic compounds. Environ. Res..

[bib0030] Varjani S.J. (2017). Microbial degradation of petroleum hydrocarbons. Bioresour. Technol..

[bib0031] Varjani S.J., Upasani V.N. (2017). A new look on factors affecting microbial degradation of petroleum hydrocarbon pollutants. Int. Biodeter. Biodegr..

[bib0032] Wang H., Lu L., Chen H., McKenna A.M., Lu J., Jin S., Zuo Y., Rosario-Ortiz F.L., Ren Z.J. (2020). Molecular transformation of crude oil contaminated soil after bioelectrochemical degradation revealed by FT-ICR mass spectrometry. Environ. Sci. Technol..

[bib0033] Wang Y.F., Zhang Z.X., Wang X.M., Guo H.X., Zhu T.T., Zhao Y.X., Lu X.B., Zhang Y.B., Ni B.J., Liu Y.W. (2023). Medium-chain fatty acids production from sewage sludge through anaerobic fermentation: a critical review. Chem. Eng. J..

[bib0034] Xu Q., Long S., Liu X.R., Duan A.B., Du M.L., Lu Q., Leng L., Leu S.Y., Wang D.B. (2023). Insights into the occurrence, fate, impacts, and control of food additives in food waste anaerobic digestion: a review. Environ. Sci. Technol..

[bib0035] Zhang L., Lin X.J., Zhang Z.F., Chen G.H., Jiang F. (2018). Elemental sulfur as an electron acceptor for organic matter removal in a new high-rate anaerobic biological wastewater treatment process. Chem. Eng. J..

[bib0036] Zhang L., Qiu Y.Y., Zhou Y., Chen G.H., Loosdrecht M.C.M.v., Jiang F. (2021). Elemental sulfur as electron donor and/or acceptor: mechanisms, applications and perspectives for biological water and wastewater treatment. Water Res.

[bib0037] Zhang L., Zhang Z.F., Sun R.R., Liang S., Chen G.H., Jiang F. (2018). Self-accelerating sulfur reduction via polysulfide to realize a high-rate sulfidogenic reactor for wastewater treatment. Water Res.

[bib0038] Zhang X.L., Li R.X., Wang J.N., Liao C.M., Zhou L.A., An J.K., Li T., Wang X., Zhou Q.X. (2022). Construction of conductive network using magnetite to enhance microflora interaction and petroleum hydrocarbons removal in plant-rhizosphere microbial electrochemical system. Chem. Eng. J..

[bib0039] Zhang Z., Lo I.M.C., Yan D.Y.S. (2015). An integrated bioremediation process for petroleum hydrocarbons removal and odor mitigation from contaminated marine sediment. Water Res.

[bib0040] Zhao Q., Liu Y., Liao C., Yan X., Tian L., Li T., Li N., Wang X. (2023). Reduction of S^0^ deposited on electroactive biofilm under an oxidative potential. Sci. Total. Environ..

[bib0041] Zhao S.N., Chen W.H., Liu M.L., Lv H.Y., Liu Y.G., Niu Q.G. (2022). Biogas production, DOM performance and microbial community changes in anaerobic co-digestion of chicken manure with and green waste. Biomass Bioenerg.

