# Supplementary material for: Dual-purpose elemental sulfur for capturing and accelerating biodegradation of petroleum hydrocarbons in anaerobic environment
Source: Water Res X. 2024 Dec 2;26:100290. doi: 10.1016/j.wroa.2024.100290 (PMC11664143; doi:10.1016/j.wroa.2024.100290)
Supplement: Supplementary file 1 [file mmc1.docx]

-Supporting Information-

**Dual-Purpose Elemental Sulfur for Capturing and Accelerating Biodegradation of Petroleum Hydrocarbons in Anaerobic Environment**

Qian Zhao ^a^, Chengmei Liao ^a, b^, Enli Jiang ^a^, Xuejun Yan ^a^, Huijuan Su ^a^, Lili Tian ^c^, Nan Li ^d^, Fernanda Leite Lobo ^e^, Xin Wang ^a^ *

^a^ MOE Key Laboratory of Pollution Processes and Environmental Criteria, Tianjin Key Laboratory of Environmental Remediation and Pollution Control, Nankai University, No. 38 Tongyan Road, Jinnan District, Tianjin, 300350, China

^b^ School of Ecology and Environment, Inner Mongolia University, Hohhot 010021, China

^c^ School of Environmental and Chemical Engineering, Yanshan University, Qinhuangdao 066004, China

^d^ School of Environmental Science and Engineering, Tianjin University, No. 135 Yaguan Road, Jinnan District, Tianjin, 300350, China

^e^ Department of Hydraulic and Environmental Engineering, Federal University of Ceará (UFC), Campus Do Pici, 60.440-900, Fortaleza, CE, Brazil

* Corresponding author: Phone: (86)18722292585; fax: (86)22-23501117; E-mail: [xinwang1@nankai.edu.cn](mailto:xinwang1@nankai.edu.cn)

**Section S1 The detailed configurations of permeable reactive barrier reactor**

The permeable reactive barrier reactor consists of a rectangular main body (15 cm × 10 cm × 10 cm) of the reactor, two peristaltic pumps, one mixing bottle (250 mL) with a magnetic stirrer, and one water stop valve for sampling, each of which was connected by fluorine hose (inner and outer diameter of 2.54 mm and 4.24 mm) with series straight joints. (**Fig. 1A**)

**Section S2 The detailed preparation method of petroleum-contaminated soil extract**

The petroleum-contaminated soil was naturally dried in a ventilated environment and passed through a 2 mm sieve in advance. A weight of 800 g soil was added to 1.6 L 50 mM phosphate buffer solution (**Table S1**) in a two-liter bottle. The liquid supernatant was used as the initial inoculum after mixing thoroughly and quiescence overnight.

**Section S3 The detailed extraction procedures of petroleum hydrocarbons**

Petroleum hydrocarbons were extracted from the entire enrichment medium using isopyknic dichloromethane in a sterile triangular flask. The serum bottle was then rinsed with dichloromethane to remove residues, which were also transferred to the flask. The mixture in the sealed flask was shaken on a reciprocating shaker overnight. The dichloromethane phase was separated from the water phase using a separatory funnel and then dehydrated with anhydrous Na_2_SO_4_ to remove any residual water from the dichloromethane. It was evaporated in a thermostatic water bath at 42 °C to a final volume of 1.5 mL. A sample of pure dichloromethane was prepared as the negative control. The saturated hydrocarbons and PAHs were separated by a chromatography column (from top to bottom containing 1 cm anhydrous Na_2_SO_4_, 6 cm neutral Al_2_O_3_, and 12 cm silica gel) (Zhang et al., 2022). They were eluted successively with n-hexane and a mixture of n-hexane/dichloromethane (1:1) (Peng et al., 2009).

**Table S1** Composition of 50 mM phosphate buffer solution (pH = 6.74 ± 0.03).

| **Ingredient** | **Concentration** |
| --- | --- |
| NH_4_Cl | 0.31 g/L |
| NaH_2_PO_4_ | 2.132 g/L |
| KCl | 0.13 g/L |
| Na_2_HPO_4_ | 4.576 g/L |

**Table S2** Composition of trace element solution.

| **Ingredient** | **Concentration** | **Ingredient** | **Concentration** |
| --- | --- | --- | --- |
| MgSO_4_ | 3 g/L | N(CH_2_COOH)_3_ | 2g/L |
| MnSO_4_ | 0.5 g/L | H_3_BO_3_ | 0.01 g/L |
| Na_2_MoO_4_ | 0.025 g/L | NaCl | 1 g/L |
| FeSO_4_·7H_2_O | 0.1 g/L | ZnCl_2_ | 0.13 g/L |
| CuSO_4_·5H_2_O | 0.01 g/L | CaCl_2_·2H_2_O | 0.1 g/L |
| Na_2_WO_4_·2H_2_O | 0.025 g/L | CoCl_2_·6H_2_O | 0.1 g/L |
| AlK(SO_4_)_2_·12H_2_O | 0.01 g/L | NiCl_2_·6H_2_O | 0.024 g/L |

**Table S3** Composition of vitamin solution.

| **Ingredient** | **Concentration** | **Ingredient** | **Concentration** |
| --- | --- | --- | --- |
| Biotin | 0.002 g/L | Nicotinic Acid | 0.005 g/L |
| Pantothenic Acid | 0.005 g/L | Thiamine | 0.005 g/L |
| B-12 | 0.0001 g/L | Riboflavin | 0.005 g/L |
| p-aminobenzoic acid | 0.005 g/L | Pyridoxine HCl | 0.01 g/L |
| Thioctic Acid (alpha lipoic) | 0.005 g/L | Folic Acid | 0.002 g/L |

**Table S4** The concentration of sulfur-related species.

| S species | Sulfide  (S^2−^, HS^−^, H_2_S)  mM | Sulfate  (SO_4_^2−^)  mM | Thiosulfate  (S_2_O_3_^2−^)  mM | Polysulfide  (S_n_^2−^, OD_600_)  1 | Sulfite  (SO_3_^2−^)  mM |
| --- | --- | --- | --- | --- | --- |
| *C*_i_ | 0.26±0.03 | 8.9±0.1 | 0.003±0.001 | 0.45±0.01 | 0.086±0.003 |
| *C*_f_ | 6.5±0.9 | 20±3 | 0.093±0.0005 | 0.64±0.02 | 0.14±0.006 |

**
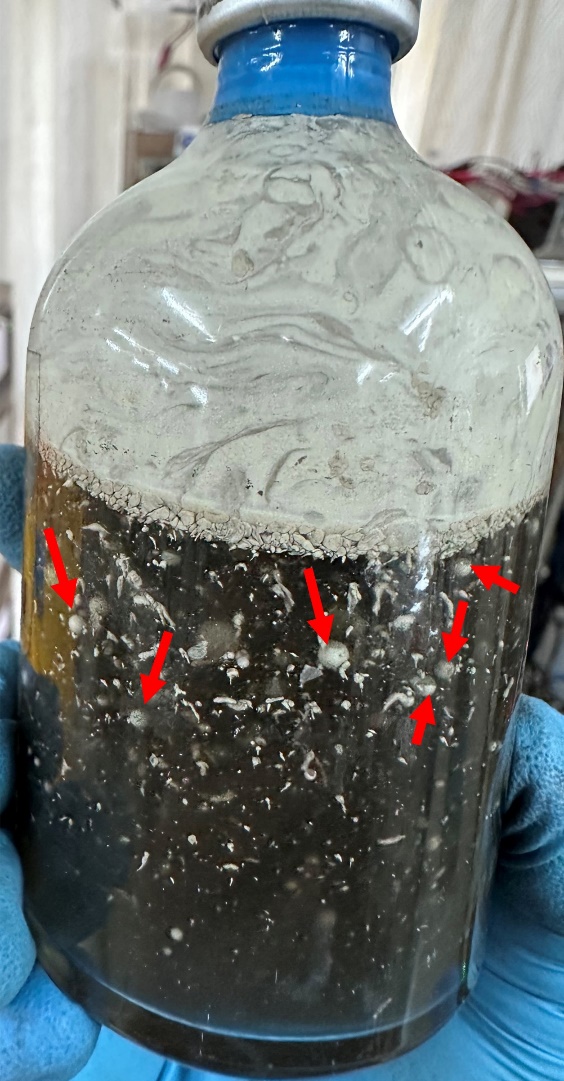
**

**Fig. S1** The magnified view of emulsification and the red arrow points to the droplets formed from the mixture of oil, water, and sulfur.

**
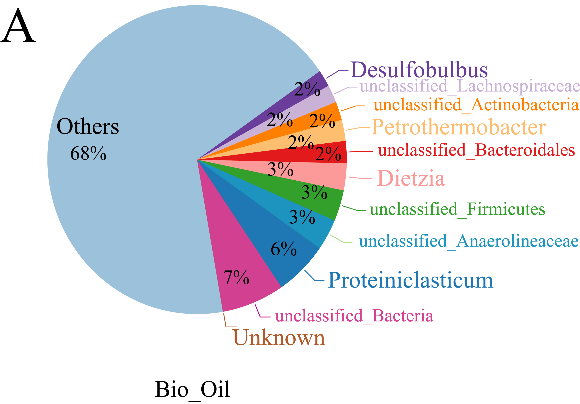

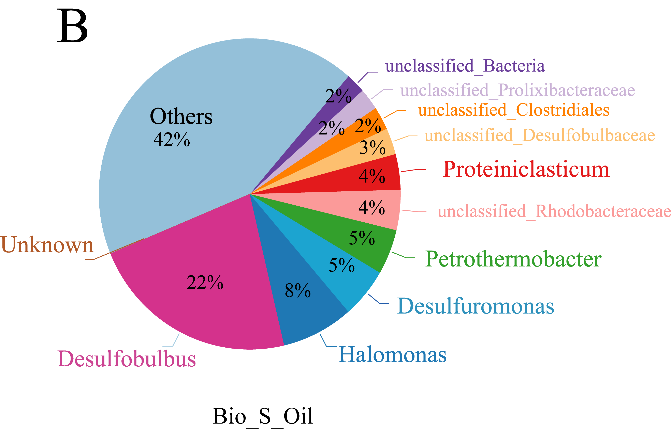
**

**
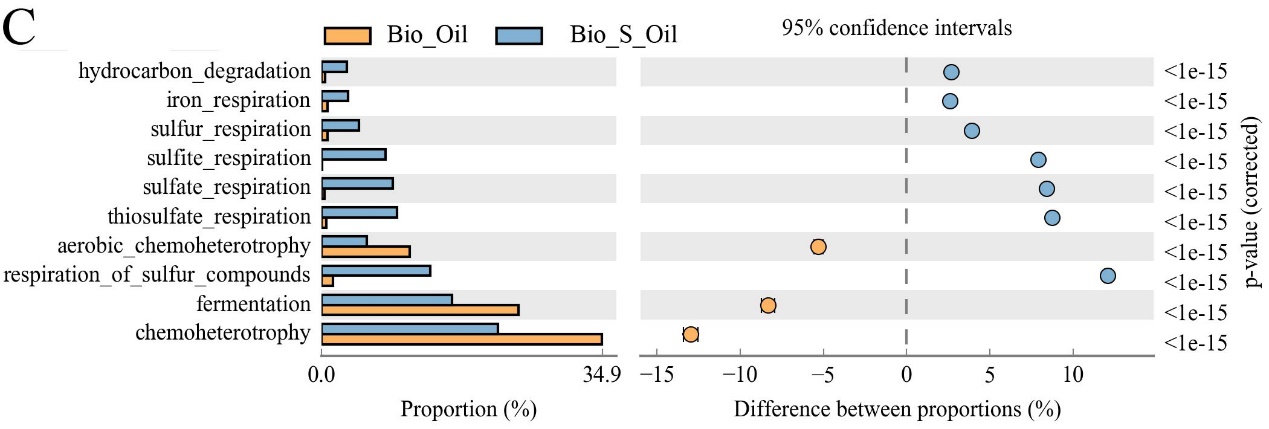
**

**Fig. S2** The microbial species (top 10 genera) in the absence of S^0^ (A, Bio_Oil) and the presence of S^0^ (B, Bio_S_Oil). Microbial functional difference analysis (C, top 10) between the Bio_Oil and Bio_S_Oil.

**Reference**

Peng, S.W., Zhou, Q.X., Cai, Z. and Zhang, Z.N. 2009. Phytoremediation of petroleum contaminated soils by Mirabilis Jalapa L. in a greenhouse plot experiment. J. Hazard. Mater. 168(2-3), 1490–1496.

Zhang, X.L., Li, R.X., Wang, J.N., Liao, C.M., Zhou, L.A., An, J.K., Li, T., Wang, X. and Zhou, Q.X. 2022. Construction of conductive network using magnetite to enhance microflora interaction and petroleum hydrocarbons removal in plant-rhizosphere microbial electrochemical system. Chem. Eng. J. 433, No. 133600.
